# A Simple Modified Technique of Pleuropericardial Window: Towards 0% Recurrence

**DOI:** 10.21470/1678-9741-2022-0017

**Published:** 2023

**Authors:** Hanan M Hemead, Amr Saleh, Wael Hassanein

**Affiliations:** 1 Cardiothoracic Surgery Department, Faculty of Medicine, Alexandria, Egypt

**Keywords:** Pericardial Effusion, Pericardial Window Techniques, Pericardiocentesis, Vena Cava, Superior

## Abstract

Recurrent pericardial effusion is commonly encountered in neoplastic and infective disorders. Intervention is compulsory in patients with unstable hemodynamics and tamponading effusion. Surgical options include: pericardiocentesis, subxiphoid pericardiostomy, and pericardial window. The latter has proved to have lower incidence of recurrence; however, the technique has been continuously refined to improve the recurrence-free survival and decrease postoperative morbidity. We herein present a novel simple modification to minimize recurrence by anchoring the free edges of pericardial fenestration overlying the superior vena cava and right atrium to the chest wall. Follow-up showed no recurrence compared to 3.5% in the conventional procedure.

## INTRODUCTION

Recurrent pericardial effusion is a debilitating condition for patients with chronic pathologies such as malignancy and autoimmune disorders. Rapid accumulation will shortly compromise hemodynamics and progress to tamponading collection, heart failure, and obstructive shock. In recurrent effusions, the main aim of management is not only to decompress the pericardium but also to decrease the future recurrence as the underlying etiologies are chronic or refractory to treatment. The optimal management with a relatively low recurrence rate is pericardial window. With thoracoscopic pleuropericardial window, better exposure is achieved allowing for the removal of sufficient pericardium to form a wider durable window and management of concomitant pleural pathologies^[1,2]^. However, the incidence of recurrence with this approach is reported in up to 12% of cases^[3,4]^.

## TECHNIQUE

**Fig. 1:**
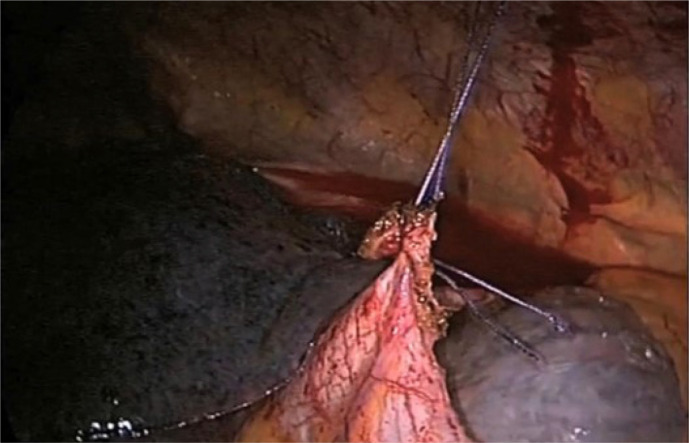
The free edge of pericardium in front of the right side of the heart is sutured to the chest wall.

A right-sided uniportal video-assisted thoracoscopic surgical approach was performed as usual. The modification entails anchoring of the free edges of pericardium overlying the superior vena cava and right atrium to the chest wall with VICRYL® sutures, creating a tent-like curtain as demonstrated in the Video 1 and Figure 1. This technique will prevent future adhesions between the edges of the pericardium, leading to a permanent wide connection between the pericardium and pleura cavities, with subsequent practical elimination of any possibility of recurrence.

**Video 1:**
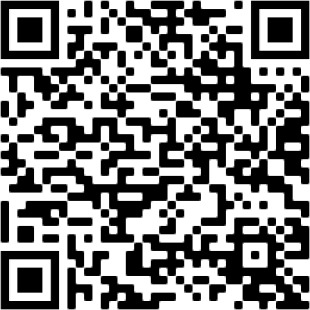
Suturing of the free pericardial edge to the anterior chest wall to minimize chances of recurrence during the procedure of video-assisted thoracic surgery pleuropericardial window for a malignant pleural effusion.

The pericardium was grasped, divided starting from the superior pulmonary veins down to the diaphragm till a satisfactory window of at least 4×4 cm is established. The phrenic nerve was being visualized throughout the procedure. The sucker was used to drain any collection and to break down any loculations. We have applied this technique for 38 patients with recurrent pericardial effusion for various pathologies during a three-year period. The mean follow-up duration was 1.2 +/- 1 year. Recurrence was not reported in any of the patients compared to a 3.5% recurrence in individuals who underwent the conventional technique previously.

## DISCUSSION

Recurrent pericardial effusion is burdensome for patients and surgeons particularly in patients with long-life expectancy. Ideally, the creation of a pleuropericardial window allows for the ongoing drainage of any effusion to the pleural space preventing the evolution of tamponading effect in the settings of effusive pericarditis. It is hypothesized that effective drainage achieved by a generous persistence window will allow for the development of symphysis and subsequent adhesions between the epicardium and the overlying pericardium. Therefore, early complete evacuation of the effusion and establishment of a persistent drainage will potentially eliminate the chances of recurrence by facilitating the development of adhesions due to inflammation and surgical trauma^[5]^. This suggests that the reported recurrence encountered in the standard procedure is due to early failure of the window to survive long enough to drain the collection adequately to facilitate the coaptation of the epicardium and overlying remaining pericardium. Therefore, the modified technique allows continuous drainage in chronic conditions. Notably, in such cases, the pericardium is stiff and non-stretchable, therefore, even a small effusion might result in hemodynamic collapse^[6]^.

## CONCLUSION

The modified technique allows for formation of a sustained orifice that serves for initial efficient removal of the collection and persistent efflux of ongoing effusion in patients with chronic conditions and expected long survival. Therefore, this technique makes the virtual odds of relapse almost approaching zero.
